# Assessment of ultrasound shear wave elastography: An animal ex‐vivo study

**DOI:** 10.1002/acm2.13924

**Published:** 2023-02-02

**Authors:** Xiuming Wang, Jiaan Zhu, Yiqun Liu, Wenxue Li, Si Chen, Huabin Zhang

**Affiliations:** ^1^ Department of Ultrasound Peking University People's Hospital Beijing People's Republic of China; ^2^ Department of Ultrasound Beijing Tsinghua Changgung Hospital School of Clinical Medicine Tsinghua University Beijing People's Republic of China

**Keywords:** lesion size, rectangular sampling box, shear wave elastography

## Abstract

**Objectives:**

To explore the influence of the surrounding environment of the target tissue, lesion size, and rectangular sampling box size on shear wave speed (SWS).

**Methods:**

The tendon SWS was acquired ex‐vivo. Then the tendons were dissected and buried in the couplant (gel) and evaluated by two‐dimensional shear wave elastography (2D‐SWE). Finally, the tendons were placed in the isolated muscles to simulate the intramuscular lesions, and their elasticity was tested under two rectangular sampling box conditions. The isolated complete liver SWS was acquired. Similarly, the large and small pieces of livers were cut out, placed in the muscles, and assessed by SWE under two rectangular sampling box conditions. The SWS acquired under different conditions was compared. Variability was evaluated using the coefficient of variation (CV). The intraclass correlation coefficient (ICC) was used to evaluate repeatability.

**Results:**

The SWS of the tendons *ex‐vivo*, buried in the couplant and placed in the isolated muscles showed significant differences (*p* < 0.001). The *ex‐vivo* condition produced the highest SWS and CV values. There were significant differences in SWS of livers with different sizes placed in muscles (*p* < 0.001). The highest SWS value was associated with small pieces of livers. No significant difference was found in SWS acquired under different rectangular box sizes (*p* > 0.05).

**Conclusions:**

Under the present study conditions, the surrounding environment of the target tissue makes a big difference to lesion SWS values. The lesion size will affect the assessment of its inherent elasticity. The size of the sampling frame has no significant effect on the tissue SWS.

## 1 INTRODUCTION

Musculoskeletal diseases such as inflammation, tumors, trauma can change the elastic properties of tissues.^1^ A clear diagnosis and timely treatment are critical to the recovery of diseases and improvement of patients' quality of life. Imaging methods such as computed tomography (CT), magnetic resonance imaging (MRI), and conventional ultrasound are available for diagnostic purposes. Two‐dimensional shear wave elastography (2D‐SWE) is a non‐invasive technology that can evaluate tissue stiffness qualitatively and quantitatively, and is expected to become an effective assessment method for evaluating the musculoskeletal system properties.^2^, ^3^, ^4^ Musculoskeletal lesions may change the biomechanical properties of tissue. SWE has the potential to become an imaging indicator that can diagnose and detect diseases non‐invasively. The feasibility of SWE in evaluating breast, liver, thyroid, and prostate diseases has been received wide acceptance.^5^, ^6^, ^7^, ^8^ However, its value in evaluating musculoskeletal system diseases still requires further research.

When applying 2D‐SWE to evaluate the elasticity of the musculoskeletal system, the clinical situation is complex and changeable. For example, muscles and tendons of different parts are adjacent to different structures, such as fat and various bones. For lesions of the same histopathology, their elasticity values might be different due to different lesion sizes. During 2D‐SWE imaging, the operator can adjust the size of the rectangular sampling box, which is a rectangular box displaying the tissue stiffness in the elastogram using a semitransparent color overlay, according to different clinical scenarios. However, systematic investigations of the influence of factors such as adjacent structures, lesion sizes and rectangular box sizes on the musculoskeletal system measurements are insufficient. Caroline Ewertsen et al. examined the healthy volunteers' biceps brachii, gastrocnemius, and quadriceps muscles with SWE in regions located above the bone and beside bone. They reported that the shear wave speed (SWS) was significantly different if the region of interest (ROI) was located above or beside bone.^9^ Skerl et al. evaluated the influence of the lesion diameter (diameter < 1.5 cm and diameter≥1.5 cm) on the diagnostic performance of 2D‐SWE of solid breast lesions and reported that the benign/malignant threshold was higher with increasing lesion size.^10^ These studies pointed out the influencing factors that may affect the SWS measurement values, but further research is needed because the research factors of previous studies were single, and there were limited studies on the musculoskeletal system. In vivo assessment of the musculoskeletal system elasticity is susceptible to many factors such as body posture, muscle state, adjacent structures, and so on. Therefore, the present study applied isolated tissue as the research object, focusing on simulating different clinical scenarios to investigate the influence of the surrounding tissues, the size of the simulated “lesion” itself, and the size of the rectangular box on SWS.

## 2 MATERIALS AND METHODS

### 2.1 Subjects

Forty‐two pieces of fresh muscles from pork tenderloin, three fresh trotters, and six complete fresh livers provided by the same slaughterhouse for consumption were chosen. These isolated fresh tissues were removed from the healthy living bodies within 6–15 h and were examined at 20°C. The average size of each muscle sample was about 15 cm × 7 cm × 7 cm with the clear muscle fiber. The study was approved by the institutional ethics board of Peking University People's Hospital.

### 2.2 Equipment

2D‐SWE were achieved by using Aixplorer system (SuperSonic Imagine, Aix‐en‐Provence, France) which employed an SL10‐2 high‐frequency linear probe. In the present study, the measurements were recorded in SWS (m/s) considering the anisotropy of muscles, because Young's modulus (kPa) has been validated only in isotropic and homogeneous tissues.^11^ The relationship between SWS and Young's modulus is E = 3ρV^2^, in which E represents Young's modulus, 3 is a constant related to Poisson's ratio for strain, ρ is tissue density (assumed to be 1 g/cm^3^), and V is the shear wave speed.^12^ The Q‐Box with a diameter of 0.1 cm was chosen. During imaging using the present system, the SWS measurement should avoid fence artifacts, signal missing areas, and areas with too high/low signal. SWE operations were performed by a sonographer with more than 3 years of experience in ultrasound elastography. The operator must be trained on the specimen to complete experimental operations at least 30 times successfully. The reading was repeated three times for each acquisition condition (including the tendons measured in trotter, the tendons measured in couplant, the tendons measured in muscle, the livers measured isolatedly, and the livers measured in muscle) with each time recorded one measurement value, and the probe was removed and replaced each time.

### 2.3 Shear wave elastography of tendon

#### 2.3.1 SWS measurement of tendons ex‐vivo

**FIGURE 1:**
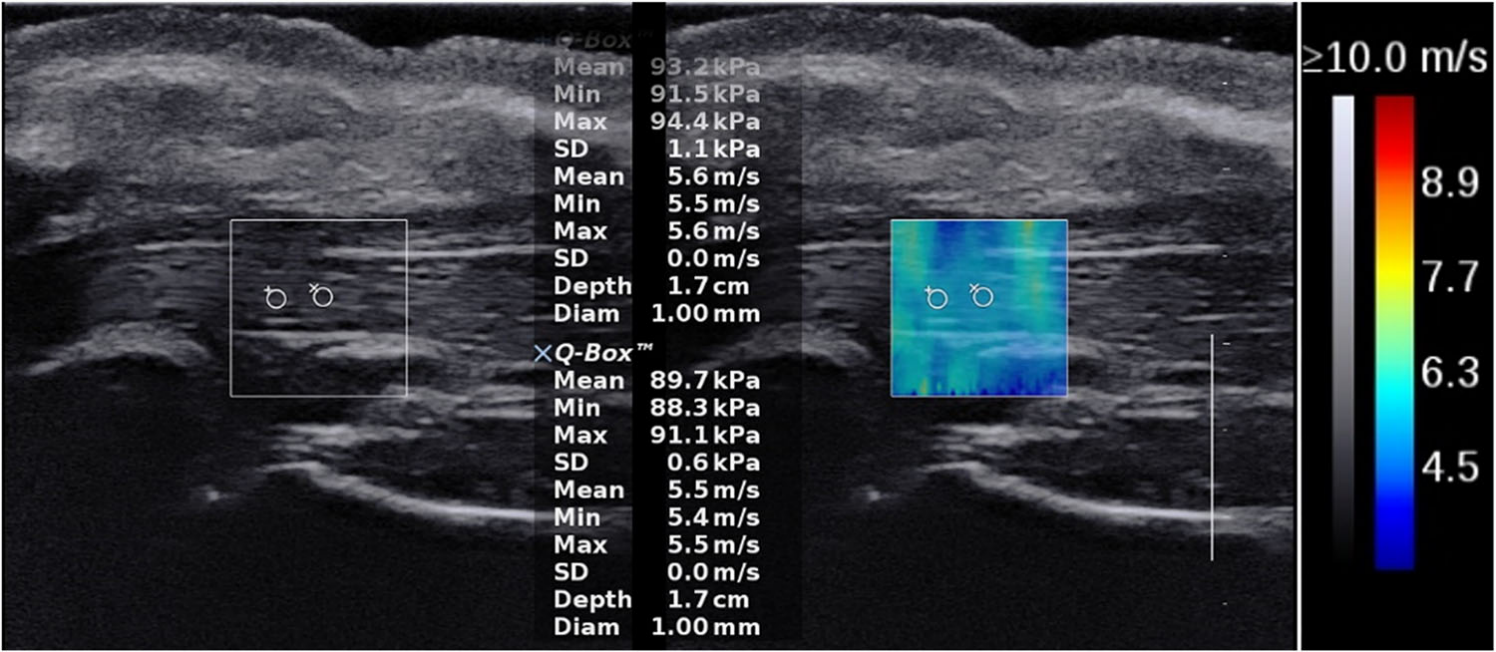
Shear wave elastic images of the tendons inside trotter.

Three fresh trotters were chosen and the third and fourth tendon branches of the flexor disitorum profundus of each trotter were found under the guidance of B‐mode ultrasound. The measurement range of each branch was from about 1 cm after the bifurcation of the two tendon branches to about 1 cm before the end of the tendon (Figure S1). Each tendon branch was divided into five parts for measurement, and the length of each part was about 1 cm. Totally six tendons (i.e., total 30 parts) were provided for measurement. The reading was repeated three times for each part. Therefore, 90 SWS measurement values were recorded in total. The rectangular box with a size of 1.0 × 1.0 cm was chosen (Figure 1).

#### 2.3.2 SWS measurement of ex‐vivo tendons buried in couplant

**FIGURE 2:**
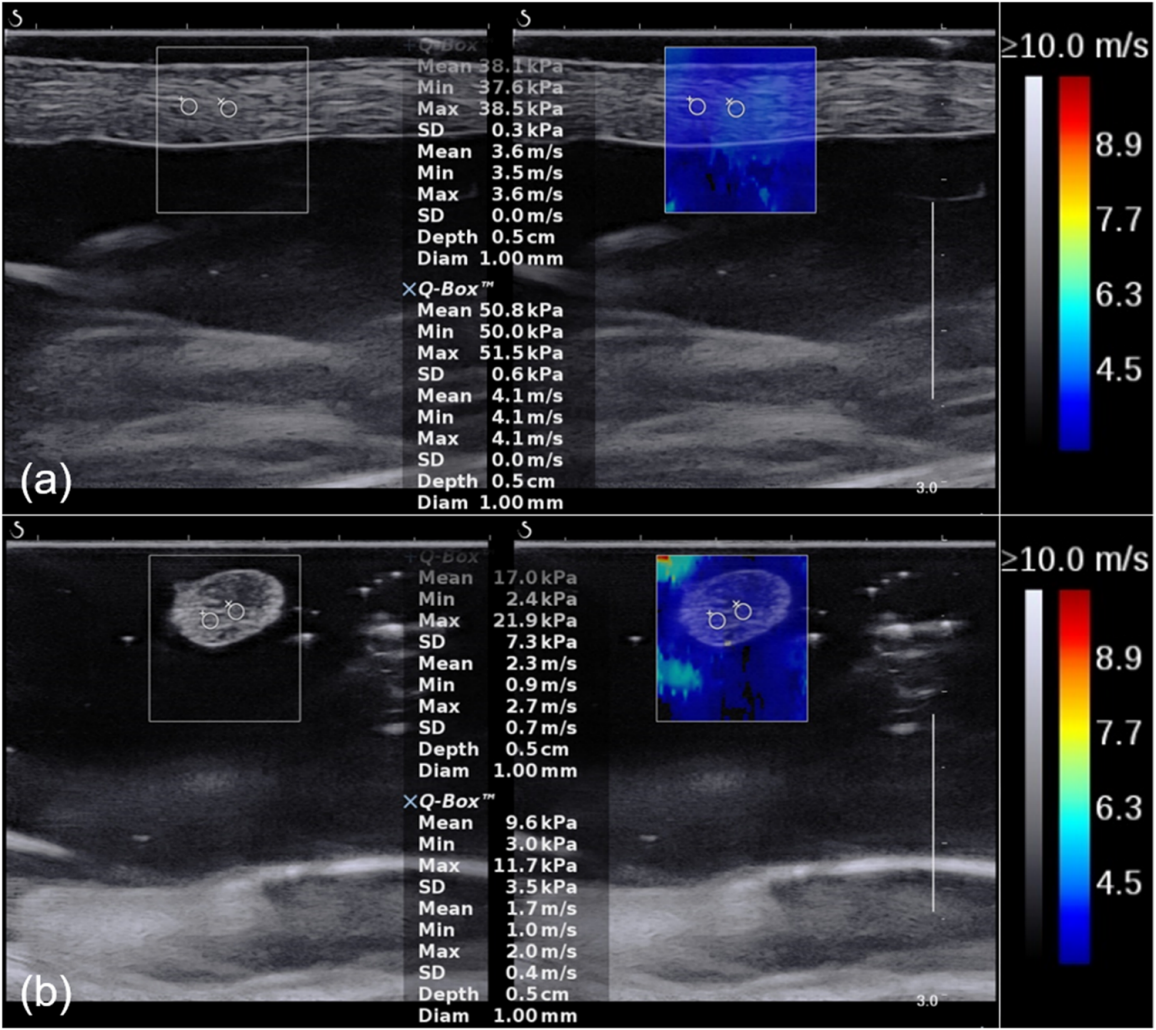
Shear wave elastic images of the tendons buried in the couplant. (a) Parallel to the muscle fiber; (b) perpendicular to the muscle fiber.

The third and fourth tendon branches of the flexor disitorum profundus of each trotter were taken out and dissected, and the measurement range was consistent with that inside trotter, marked with a marker. Then the isolated tendons were buried in a box filled with couplant. The couplant thickness above the tendon was about 0.2 cm. Similarly, totally six tendons (i.e., total 30 parts) were measured. Two probe orientations including the long axis of the probe parallel and perpendicular to the tendon fiber were chosen. The reading was repeated three times for each part. Therefore, 180 SWS measurement values were recorded in total for two orientations. The rectangular box with a size of 1.0 cm × 1.0 cm was used (Figure 2).

#### 2.3.3 SWS measurement of ex‐vivo muscles

Twenty isolated muscle samples were selected for SWE evaluation. The reading was repeated three times for each muscle. Therefore, 60 SWS measurement values were recorded in total. A rectangular box of 2.5 cm × 2.0 cm with 2 cm acquisition depth and 0.2 cm couplant thickness was applied as an acquisition method. Before the examination, a marker was used to record the specific measurement location of each sample. During imaging, the muscle specimen was placed between two fixators to maintain the shape of the muscle, but no pressure was applied.

#### 2.3.4 SWS measurement of ex‐vivo tendons embedded in the muscle

**FIGURE 3:**
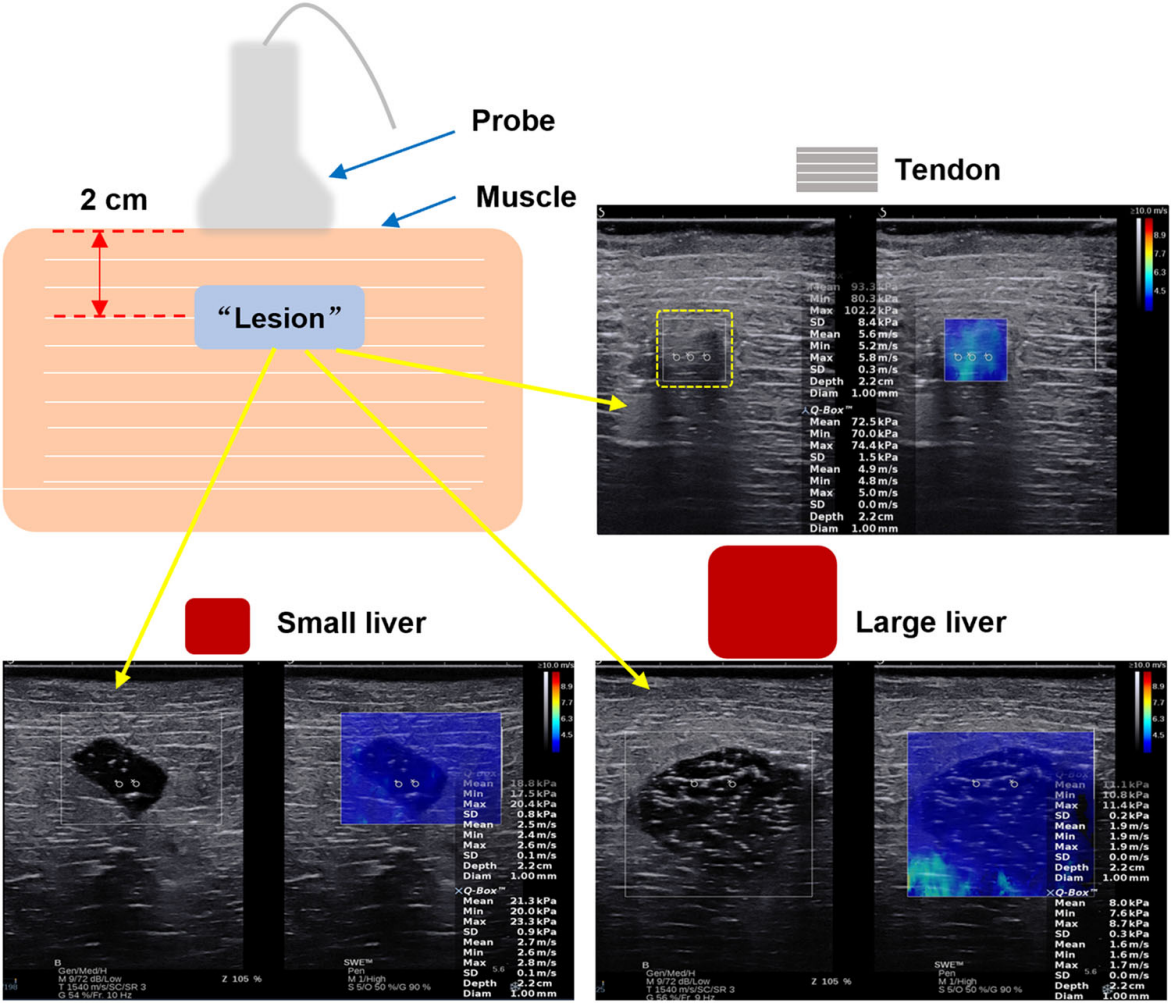
Schematic diagram of the SWS acquisition process for “lesion 1” (tendon), “lesion 2” (small liver) and “lesion 3” (large liver).

Each tendon was evenly divided and cut off into five segments. 30 segments were acquired with a 1 cm length for each segment in total. Each tendon segment was put into the muscle samples (The placement position was as close as possible to the SWS measurement position of the muscles). Each muscle sample contained one or two tendon segments (ten for one and ten for two), and the depth of the tendon in muscle was about 2 cm with the same tendon and muscle fiber direction. The embedded tendons simulated intramuscular lesions, which were defined as “lesion 1” in this study (Figure 3). Rectangular box sizes of 2.0 cm × 2.5 cm (large rectangular box) and 1.0 cm × 1.0 cm (small rectangular box) were both used for “lesion 1” SWS measurement. Therefore, 20 muscle samples contained 30 “lesions 1” (tendon segments). The reading was repeated three times for each “lesion 1” (tendon segment). 180 SWS measurement values were recorded in total because of the two different rectangular box sizes. At the same time, the muscle next to (about 1 cm) “**lesion 1”** was also measured with the rectangular box of 2.5 cm × 2.0 cm. The target tissue was placed in the middle of the rectangular box during measurement.

### 2.4 Shear wave elastography of livers

#### 2.4.1 SWS measurement of complete ex‐vivo livers

The livers were laid flat on the examining couch (with visceral surfaces of the liver downward) and a total of 22 areas were found under ultrasound guidance (avoided large bile duct and blood vessels). The surface contours of each area were outlined using a maker. Rectangular box sizes of 2.0 cm × 2.5 cm were used for liver SWS measurement. The reading was repeated three times for each area. Therefore, 66 SWS measurement values were recorded in total. Then the 22 areas of each liver were dissected out and each area was cut into two pieces (Small pieces were 1 cm × 1 cm × 1 cm and large pieces were 2 cm × 2 cm × 2 cm approximately). A total of 44 pieces including small and large pieces were obtained.

#### 2.4.2 SWS measurement of ex‐vivo lumpy livers embedded in the muscle

Twenty‐two isolated muscle specimens were selected. Each piece of lumpy liver was embedded in the isolated muscle. Each muscle sample contained two pieces of lumpy livers (a small one and a large one), and the depth of livers in muscle was about 2 cm. Therefore, 22 muscle samples contained 44 pieces of lumpy livers in total (22 small pieces and 22 large pieces). The embedded lumpy livers simulated intramuscular lesions, which were defined as “**lesion 2”** (small liver) and “**lesion 3”** (large liver) in this study. Rectangular box sizes of 3.0 cm × 3.0 cm (large rectangular box) and 1.0 cm × 1.0 cm (small rectangular box) were used for “**lesion 2”** and “**lesion 3”** SWS measurements (Figure 3). The reading was repeated three times for each piece of lumpy liver. 264 SWS measurement values (132 for small pieces and 132 for large pieces) were recorded totally under two different rectangular box sizes.

### 2.5 Data and statistics

SPSS software (version 25.0) was used for statistical analysis. All data were expressed as mean ± standard deviation (SD). *p* < 0.05 was considered statistically significant.

For the comparison of variance in the different measurement conditions, the coefficient of variation (CV) was calculated by applying the equation: CV = standard deviation/mean value. The larger the CV value, the lower the reliability and repeatability of each measurement. The Kolmogorov‐Smirnov test was used to check if the data were normally distributed. The test for homogeneity of variance was applied to check for differences in variance. One‐way ANOVA (analysis of variance) was used to compare the SWS of tendons under different acquisition methods. For comparison of two groups of data, according to the results of Kolmogorov‐Smirnov test, T‐test for the normal distribution data and Mann‐Whitney U test for not. T‐test or Mann‐Whitney U test was applied to compare SWS between different acquisition methods for *ex‐vivo* muscles, acquisition directions for tendon and “lesions”, and the rectangular box of different sizes. The intra‐class correlation coefficient (ICC) was used to assess the reliability of the measurements obtained with different rectangular box sizes and acquisition directions, and the ICC value was interpreted as follows: 0.00–0.20 for poor agreement, 0.21–0.40 for fair agreement, 0.41–0.60 for moderate agreement, 0.61–0.80 for substantial agreement, and >0.80 for an almost perfect agreement.^13^

## 3 RESULTS

### 3.1 SWS comparison of tendons and muscles under different acquisition conditions

**FIGURE 4:**
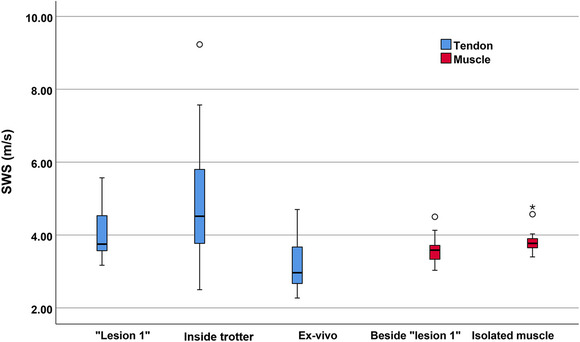
SWS comparison of the tendons and muscles under different acquisition conditions (“lesion 1”, tendon in muscle).

The SWS results of tendons and muscles under different measurement conditions for the present ultrasound system are shown in Table 1. The results showed that the tendon SWS varied with different acquisition conditions, including being placed in the muscle (“lesion 1”), inside trotter status and being buried in the couplant. The inside trotter status produced the highest SWS values and CV, while being buried in the couplant produced the lowest SWS values (Figure 4). When the tendons were inserted into the muscle tissue, there was a decrease and significant difference in SWS in muscle tissue 1 cm from the **“lesion 1”** (Figure 4).

**TABLE 1:** The SWS results of the tendons and muscles under different acquisition conditions

	Tendon			Muscle	
SWS (m/s)	“Lesion 1”	inside trotter	*Ex‐vivo (buried in the couplant)*	Muscle beside “lesion 1”	Isolated muscle
Mean ± SD	3.96 ± 0.62	4.81 ± 1.56	3.17 ± 0.62	3.57 ± 0.34	3.84 ± 0.32
CV	0.16	0.34	0.20	0.10	0.08
P	<0.001			0.007	

M ± SD, mean ± standard deviation.

### 3.2 SWS comparison of livers with different sizes

**FIGURE 5:**
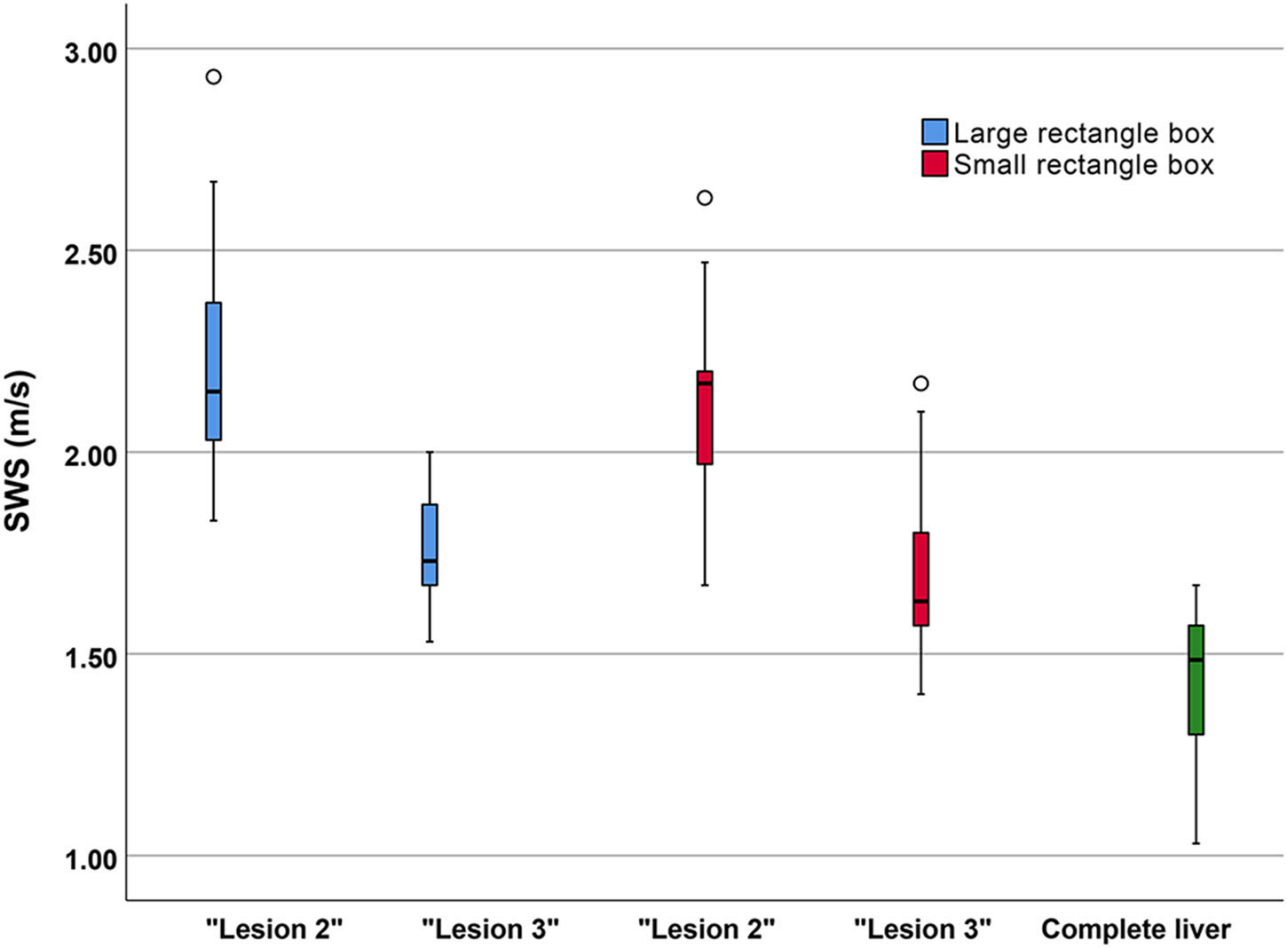
SWS comparison of the livers under different statuses (“lesion 2”, small liver; “lesion 3”, large liver).

Table 2 showed the SWS results of the complete isolated liver, “lesion 2” (small liver) and “lesion 3” (large liver) under different rectangular box sizes for the present ultrasound system. The results showed that the SWS values of “lesion 2” were higher and significantly different from “lesion 3” (Figure 5). The SWS values of “lesion 2” and “lesion 3” were both higher than the intact isolated liver (Figure 5).

**TABLE 2:** The SWS results of the livers under different status

	Large rectangular box			Small rectangular box				
SWS (m/s)	“Lesion 2”	“Lesion 3”	*p*	“Lesion 2”	“Lesion 3”	*p*	Complete liver	*p*
Mean ± SD	2.22 ± 0.28	1.76 ± 0.13	<0.001	2.10 ± 0.23	1.72 ± 0.21	<0.001	1.44 ± 0.17	<0.001
CV	0.13	0.07		0.11	0.12		0.12	

M ± SD, mean ± standard deviation; “lesion 2”, small liver; “lesion 3”, large liver.

### 3.3 SWS comparison of “lesions” between different rectangular box sizes

**FIGURE 6:**
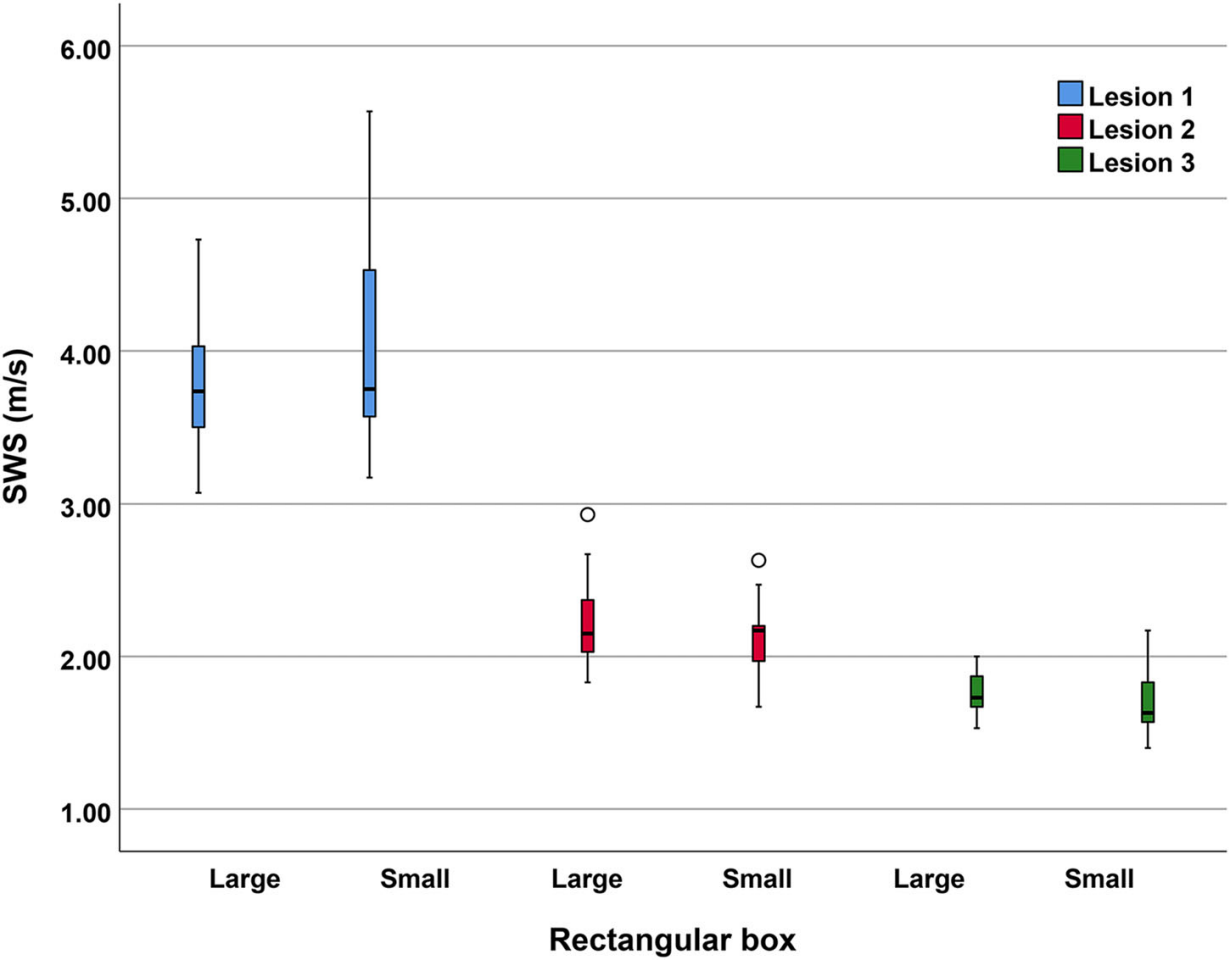
SWS comparison of the three “lesions” under different rectangular boxes.

The SWS results of using the large and small rectangular box for the present ultrasound system were shown in Table 3. The results showed that there was no significant difference in the SWS obtained under different rectangular box sizes for “lesion 1”, “lesion 2” and “lesion 3”. Figure 6 showed the SWS comparison results of “lesion 1”, “lesion 2” and “lesion 3” under different rectangular box sizes.

**TABLE 3:** The SWS results of different tissues with different rectangular box

	“Lesion 1”			“Lesion 2”			“Lesion 3”		
SWS (m/s)	Large	Small	*p*	Large	Small	*p*	Large	Small	*p*
Mean ± SD	3.81 ± 0.42	3.97 ± 0.62	0.24	2.22 ± 0.28	2.11 ± 0.23	0.15	1.76 ± 0.13	1.72 ± 0.21	0.42
CV	0.11	0.16		0.13	0.11		0.07	0.12	
ICC	0.46(0.24–0.67)	0.71(0.55–0.84)		0.28(0.02–0.56)	0.15(0–0.45)		0.17(0–0.46)	0.43(0.17–0.68)	

M ± SD, mean ± standard deviation; “lesion 1”, tendon; “lesion 2”, small liver; “lesion 3”, large liver; large, large rectangular box; small, small rectangular box.

### 3.4 SWS comparison of tendons between different acquisition directions

Table 4 summarizes the tendon SWS under two different acquisition directions (along the fiber and perpendicular to the fiber). The results showed that the SWS of the tendons measured in two directions were significantly different. The SWS obtained along the fiber direction was higher than that in the vertical direction, along with higher ICC and better reliability.

**TABLE 4:** The SWS results of the tendons under two acquisition orientations

	SWS (m/s)		
Direction	Mean ± SD	CV	ICC
Parallel	3.17 ± 0.62	0.20	0.91(0.84–0.95)
Vertical	2.80 ± 0.57	0.20	0.39(0.04–0.66)
*p*	0.02		

M ± SD, mean ± standard deviation.

## 4 DISCUSSION

Musculoskeletal lesions are very complex and diverse, including inflammation, tumors, trauma, et al. Early detection and definitive diagnosis are critical to patients' quality of life and prognosis. The current imaging methods mainly include MRI, CT and ultrasonography. However, MRI is expensive, and time‐consuming, while the soft‐tissue resolution of CT is low. SWE technology, which can provide tissue anatomical and elastic information with low cost, is expected to become an effective evaluation method for musculoskeletal lesions.

The application of SWE in the musculoskeletal system field still needs further research. It is necessary to understand the anatomical structure of the tissue, the basic biomechanical properties, and the limitations of the SWE technology, as many technical and anatomical factors will affect the elasticity measurement of the musculoskeletal system.^14^ Various adjacent tissues and complex muscle structures make it difficult to evaluate the elasticity of the musculoskeletal system. In the present study, firstly the SWS changes of muscle or tendon tissue in the different surrounding environments *ex‐vivo* were explored. Secondly, the simulated lesions in muscle tissue *ex‐vivo* were made to evaluate the influence of the “lesion” size on the SWS values. At the same time, the influence of different rectangular box sizes on the SWS values of the tissue was also discussed.

Currently, there are limited data on the influence of the surrounding tissue environment on the tested tissue elasticity. In the present study, the SWS exhibited a significant difference when the tendon was inside trotter, placed in the couplant and the isolated muscle tissue. As a result, the SWS values would be different for the tissue of the same histopathological structure when adjoining different tissues. And it can be found that the harder the surrounding tissue, the higher the tested tissue SWS. This may be attributed to the influence of the surrounding environment on the propagation speed of shear waves in the target tissue.^15^, ^16^, ^17^, ^18^ The highest SWS value was related to the tendon inside trotter, while the lowest SWS value was related to the tendon placed in the couplant. More bones and soft tissues around the tendon inside the trotter leading to faster shear wave propagation might account for this phenomenon. The SWS value of the tendon inside the trotter has the highest CV, and the worst measurement repeatability, which was attributed to the complex tissue structure adjacent to the tendon inside the trotter resulting in inconsistent shear wave propagation speed. For the isolated muscle embedded with the tendon, that is, the muscle tissue adjacent to the “lesion 1” (1 cm away from the lesion), the SWS values were lower and significantly different compared with the muscle without embedded tendon. It is suggested that the condition of the surrounding tissues will affect the SWS measurement values of the target lesions when assessing the elasticity of the lesions. Therefore, its SWS may vary with different surrounding tissues even for the lesions of the same histopathology. Thus, in clinical practice, it may be not suitable to compare the SWS of lesions with the same histopathology but different locations. In addition, it is also necessary to consider the possible impact of the lesion on the SWS of target tissue when assessing the elasticity of the tissue around the lesion.

Previous studies have shown that the size of benign and malignant breast lesions can affect the elasticity of the lesion; the size of the lesion is positively correlated with the maximum elasticity of breast cancer.^19^, ^20^ Antonio Bulum et al. divided all lesions into small (diameter < 1.5 cm) and large lesions (diameter ≥ 1.5 cm) to investigate the effect of lesion size on the performance of SWE in differentiating malignant breast lesions, and found that larger lesions had significantly higher E_mean_ and E_max_ values.^21^ However, these studies are limited to In vivo breast lesions, and there will be histopathological differences between lesions of different sizes, which will also affect the elasticity of the tissue. In the present study, liver tissues were regarded as simulated “lesions” and liver tissues of different sizes were placed into muscle tissues *ex‐vivo*. It was found that there was a significant difference in SWS values between two almost identical lesions of different sizes. Moreover, the SWS values of the “lesion” were higher than those of the complete liver. Under the present study conditions, it indicated that the size of the “lesion” affected the SWE evaluation of “lesion” inherent elasticity. The ratio of the lesion and the surrounding tissue will affect the shear wave propagation in the tissue. The harder and larger the surrounding tissue, the faster the SWS, and the higher the “lesion” elasticity.

In the present study, three different types of “lesions” were simulated *ex‐vivo*, namely, “lesion 1” (tendon), “lesion 2” (small liver), and “lesion 3” (large liver). Three lesions were implanted into isolated muscle tissue, then underwent SWE evaluation with two rectangular boxes of different sizes. It was found that there was no significant difference between the SWS values obtained under different rectangular boxes. A rectangular sampling box was defined during imaging, and the color‐coded tissue elasticity information will be displayed in the rectangular sampling box. The SWE technology of the Aixplorer system was adopted in the present study. The high‐speed imaging mode of the system can emit ultrasonic plane waves into the tissue, thus one emission can penetrate the whole imaging plane. Therefore, the acoustic radiation force generated by the acoustic beam can be evenly distributed in the tissue with nearly no distribution difference of ultrasonic beam at each position, even rectangular boxes have different sizes. Finally, technical operations during the imaging process may also affect the SWS value.^11^, ^22^ In the present study, we always tried to place the rectangular box in the center of the visual field and the “lesion” in the center of the rectangular box. Under different rectangular box conditions, the focus of sound beam in the central “lesion” is similar, and the SWS value will also be similar. However, at present, there are few research reports on how to select the appropriate rectangular box size when applying SWE technology up to now. The results in the present study are only applicable to the present condition of the current system. In the process of clinical application, further research is needed to define the rectangular box size when comparing the elasticity of the lesion with the same histopathology but a different size.

There have been many studies on the influence of the detection direction of muscles and tendons on the tissues SWS, and the conclusions of these studies are pretty consistent, that is, it was recommended to detect with the direction parallel to the fiber.^23^, ^24^ Cortez et al. measured the SWS of the gastrocnemius and tibial anterior muscles In vivo, and found that when the detection direction was perpendicular to the fiber direction, the SWS was lower than that parallel to the fiber direction. When the detection direction was parallel to the fiber direction, the SWS measurement repeatability is higher.^23^ In the present study, the isolated tendon was used as the research object, and it was found that the SWS value obtained with the parallel direction was higher along with better reliability. The main factor that affects the tendon elasticity evaluation is the anisotropic physical properties of the tendon. The anisotropy level perpendicular to the tendon fiber is higher than that parallel to the fiber direction, and the shear wave propagates poorly in the tissue with a high anisotropy level, resulting in lower SWS measurement values and uneven elastic distribution in elastogram.^23^, ^24^, ^25^, ^26^ Therefore, the direction parallel to the tendon fiber brings in good SWS repeatability, which is in favor of accurate elasticity measurement and follow‐up of the target tissue.

## 5 CONCLUSIONS

Under the present study conditions, the SWS value of the lesion is affected by the condition of the surrounding tissues when evaluating the elasticity of the target lesion. Thus, the SWS value varied even for the same kind of disease, and the lesion could also make a difference to the elasticity evaluation of the surrounding tissue. The size of the “lesions” will affect the SWE assessment of their inherent elasticity. The higher the hardness and the larger the ratio of the surrounding tissue, the higher the elasticity values of the “lesion” will be. Under the present conditions, the size of the rectangular box has no significant effect on the SWS value for the same lesion. The size of the rectangular box can be adjusted according to different clinical scenarios. The detection direction parallel to the fiber is recommended when using SWE technology to evaluate the tendon elasticity, which has higher SWS values and better reliability.

This work has some limitations. First, the sample size was not large enough. Second, the present work mainly applied *ex‐vivo* research methods. The elasticity of biological tissues might change with time. Moreover, the temperature and humidity of the *ex‐vivo* tissue were different from those in the body and the mutual embedding between different tissues might affect the elasticity of the tissue.

## AUTHOR CONTRIBUTION

Xiuming Wang: Acquisition, analysis, and interpretation of data; drafting the work; final approval of the version. Jiaan Zhu: Conception and design of the work; revising the manuscript; agreement to be accountable for all aspects of the work; final approval of the version. Yiqun Liu: Analysis of data; revising the manuscript; final approval of the version. Wenxue Li: Acquisition of data; revising the manuscript; final approval of the version. Si Chen: Revising the manuscript; final approval of the version. Huabin Zhang: Revising the manuscript; final approval of the version.

## CONFLICT OF INTEREST

The authors declare no conflict of interest.

## Supporting information

Supporting Information
