# Supplementary material for: Assessment of ultrasound shear wave elastography: An animal ex‐vivo study
Source: J Appl Clin Med Phys. 2023 Feb 2;24(4):e13924. doi: 10.1002/acm2.13924 (PMC10113705; doi:10.1002/acm2.13924)
Supplement: Supplementary file 1 — Supporting Information [file ACM2-24-e13924-s001.docx]

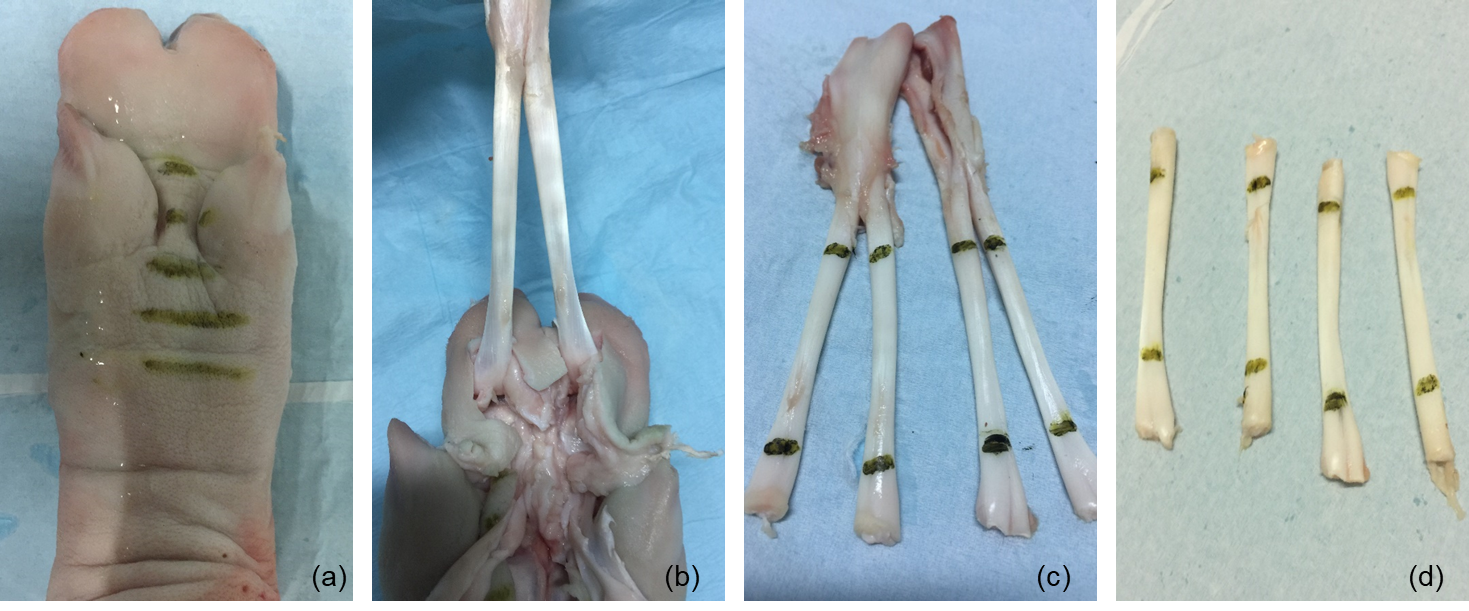


**Fig. S1** (a) SWS measurement positions of tendons “*in vivo*”; (b) - (d) The process of dissecting out the tendon and marking the measurement range with a marker.
